# Liver Fluke Induces Cholangiocarcinoma

**DOI:** 10.1371/journal.pmed.0040201

**Published:** 2007-07-10

**Authors:** Banchob Sripa, Sasithorn Kaewkes, Paiboon Sithithaworn, Eimorn Mairiang, Thewarach Laha, Michael Smout, Chawalit Pairojkul, Vajaraphongsa Bhudhisawasdi, Smarn Tesana, Bandit Thinkamrop, Jeffrey M Bethony, Alex Loukas, Paul J Brindley

## Abstract

The authors discuss the molecular pathogenesis of opisthorchiasis and associated cholangiocarcinogenesis, particularly nitrative and oxidative DNA damage and the clinical manifestations of cholangiocarcinoma.

## Opisthorchiasis and Clonorchiasis: Major Regional Public Health Problems

Liver fluke infection caused by Opisthorchis viverrini, O. felineus, and Clonorchis sinensis is a major public health problem in East Asia and Eastern Europe. Currently, more than 600 million people are at risk of infection with these trematodes [1]. O. viverrini is endemic in Southeast Asian countries, including Thailand, Lao People's Democratic Republic, Vietnam, and Cambodia [2], and C. sinensis infection is common in rural areas of Korea and China. Opisthorchiasis has been extensively studied in Thailand, where an estimated 6 million people are infected with the liver fluke (calculated from overall 9.4% prevalence within the population in 2001) [3].

**Figure 1:**
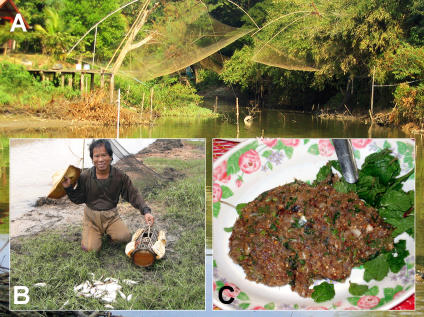
Preparation of a Meal of *Koi-Pla* Using Uncooked Cyprinoid Fishes (A) Fluke-infected fish are plentiful in the local rivers such as the Chi River in Khon Kaen province, Thailand. (B) Local people catch the fish in nets and prepare the fish-based meals with local herbs, spices, and condiments. (C) The finished dish of *koi-pla* accompanied by rice and vegetables. This dish is a dietary staple of many northeastern Thai villagers and is a common source of infection with O. viverrini.

**Figure 2:**
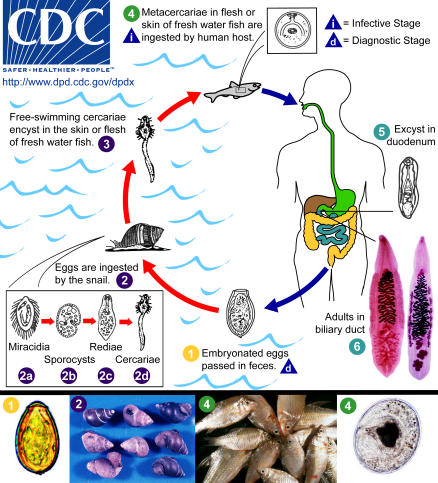
Life Cycles of O. viverrini and C. sinensis Embryonated eggs are discharged in the biliary ducts and in the stool (1). Eggs are ingested by a suitable snail intermediate host (2); there are more than 100 species of snails that can serve as intermediate hosts. Each egg releases a miracidium (2a), which goes through several developmental stages (sporocyst [2b], redia [2c], and cercaria [2d]. The cercaria is released from the snail and after a short period of free-swimming time in water, it penetrates the flesh of a freshwater fish, such as Cyclocheilichthys armatus or Puntius leiacanthus, where it encysts as a metacercaria (3). Humans are infected through ingestion of undercooked, salted, pickled, or smoked freshwater fishes (4). After ingestion, the metacercaria excysts in the duodenum (5) and ascends the biliary tract through the ampulla of Vater. Maturation to adulthood (right, O. viverrini; left, C. sinensis) takes approximately one month (6). The adult flukes (measuring 10–25 mm by 3–5 mm) reside in the small and medium-sized biliary ducts. In addition to humans, carnivorous animals can serve as reservoir hosts. (Adapted from http://www.dpd.cdc.gov/DPDx/HTML/Opisthorchiasis.htm.)

Infection with these food-borne parasites is prevalent in areas where uncooked cyprinoid fish are a staple of the diet. Due to poor sanitation practices and inadequate sewerage infrastructure, people infected with O. viverrini and C. sinensis pass parasite eggs in their faeces into natural water reservoirs, where the parasite eggs are eaten by intermediate host snails, for example, aquatic snails of the genus Bithynia, the first intermediate host of O. viverrini. After hatching, free swimming parasites, called cercariae, are released from the infected snails. Cercariae then locate their next intermediate host, cyprinoid fishes, encyst in the fins, skin, and muscles of the fish, and become metacercariae. The metacercariae are infective to humans and other fish-eating mammals upon ingestion of raw or undercooked fish in dishes such as *koi-pla* (Figure 1), and in turn the parasite's life cycle is completed (Figure 2).

Most people with opisthorchiasis or clonorchiasis have no symptoms. Only 5%–10% of infected people, in general those with heavy fluke infections, have non-specific symptoms such as right upper quadrant abdominal pain, flatulence, and fatigue [4,5]. Enlargement of the gall bladder can be detected by ultrasonography, and is reversed after elimination of flukes by praziquantel [6]. Nonetheless, heavy, long-standing infection is associated with a number of hepatobiliary diseases, including cholangitis, obstructive jaundice, hepatomegaly, fibrosis of the periportal system, cholecystitis, and cholelithiasis [7–10]. Moreover, both experimental and epidemiologic evidence strongly implicates liver fluke infection in the aetiology of one of the liver cancer subtypes—cholangiocarcinoma (CCA), or cancer of the bile ducts [2,11].

The pathology of clonorchiasis was recently reviewed in detail by Rim [12]. Unlike O. viverrini, C. sinensis is not considered a Group I carcinogen (known to be carcinogenic in humans) [2], despite its widespread prevalence [13]. In this review we therefore focus on opisthorchiasis, where the link between fluke infection and CCA is more robust than for clonorchiasis. Further, the article reviews the molecular pathogenesis of opisthorchiasis and associated cholangiocarcinogenesis, particularly nitrative and oxidative DNA damage and clinical manifestations of CCA that were either not addressed or available in our previous review [14].

## Liver Fluke Infection and Cholangiocarcinoma

Second to tobacco use, infections are the most important preventable source of human malignancies [15–17] (Table 1). The association between the occurrence of CCA and the presence of liver flukes has been known for about 50 years [18].

**Table 1:**
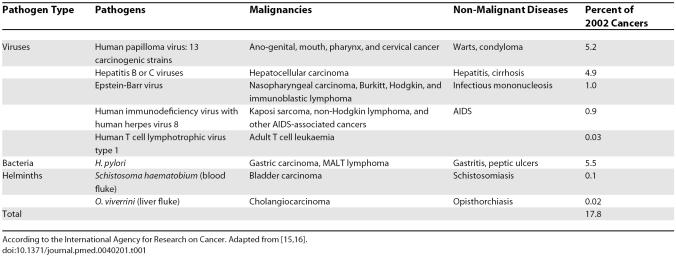
Malignancies Attributable to Infectious Agents

**Figure 3:**
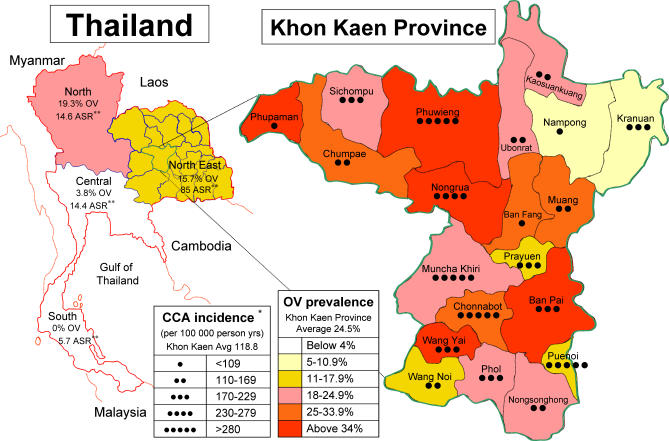
Incidence of CCA and O. viverrini in Thailand from 1990–2001 Increasing intensity of red represents increasing prevalence of O. viverrini, while increasing number of dots represents increasing cancer rates. In general, higher O. viverrini prevalence correlates with a higher CCA burden, although sporadic anthelmintic therapy has influenced this relationship. It should be noted that even one spot represents significant cancer rates anywhere else in the world. *Truncated age-standardised incidence from 35–64 years. **Age-standardised incidence of CCA throughout registered regions. Adapted from [20].

In Thailand, the first evidence for such an association came from hospital case series. CCA was observed in a high percentage of liver cancers from northeast Thailand, where the prevalence of O. viverrini infection is higher than elsewhere in this country [19]. More formal correlation studies showed that the incidence of CCA in the five major regions of Thailand varied by at least 12-fold and had a strong positive correlation with the prevalence of O. viverrini infection, as measured by anti-O. viverrini antibody titres in the general population (Figure 3) [20,21]. Similar correlations have been observed in populations in Laos [22,23]. Hepatocellular carcinoma (HCC) shows no such relationship.

Several similar surveys of Thai villagers showed a strong association between the intensity of O. viverrini infection, parasite-specific antibody response, and abnormalities of the biliary tract, including suspected CCA [8,24]. A linear trend of the frequency of suspected CCA and O. viverrini faecal egg count was observed, where an odds ratio of 14.1 was found in a group with elevated egg counts [25]. Moreover, elevated anti-O. viverrini antibody titres increase the risk of appearance of CCA by up to 27-fold [26].

The geographical pattern of liver fluke infection in Thailand is not uniform, with the greatest prevalence occurring in the north (19.3%) and northeast (15.7%), compared with the central (3.8%) and southern regions (0%) [3]. Khon Kaen province in northeast Thailand has the highest incidence of this type of cancer in the world [27,28].

In Thailand, despite widespread implementation of chemotherapy with praziquantel in the past, the prevalence of O. viverrini in some endemic areas approaches 70%. Moreover, in Thailand, liver cancer is the most prevalent of the fatal neoplasias, and rates of CCA in regions where the parasite is endemic are unprecedented. By way of comparison, CCA is responsible for about 19% of liver cancers in the United States, compared with 71% in Khon Kaen, Thailand, representing the highest incidence of CCA in the world in this region [28].

## Clinical Manifestations

Most primary malignant liver cancers are of two main histologic types distinguished by their cellular origin. HCC derives from hepatocytes, the main cells in the liver, and is the most common form of liver cancer throughout the world. There are several important risk factors that have been demonstrated with varied mechanisms of action; 75%–80% of cases are attributable to hepatitis B and C infection [29,30]. CCA is derived from cholangiocytes, which form the epithelial lining of both intrahepatic and extrahepatic bile ducts, except for those of the gallbladder. This form of liver cancer has a lower operable rate than HCC and most cases are currently untreatable except for complete liver transplantation [31]. Compared to HCC, CCA is a considerably less common form of liver cancer, except in regions within East Asia where infection with O. viverrini, and to a lesser extent, C. sinensis, is widespread [32].

**Figure 4:**
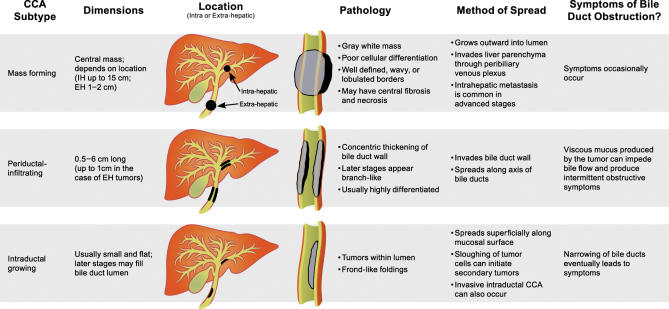
Characteristics of CCA Morphologically this cancer falls into three subtypes: mass forming, periductal infiltrating, and intraductal. This classification is independent of the location of the affected bile duct, which may be intrahepatic (within the liver) or extrahepatic (outside the liver, excluding the gallbladder). The line between these subtypes tends to blur as the malignancy matures. Tumour dimensions, mode of spreading, and clinical manifestations are noted. Adapted from [36,37] by Giovanni Maki.

CCA represents a formidable diagnostic and treatment challenge [33]. The wide variation in CCA biology and the rarity of the condition (which represents only 3% incidence of all gastrointestinal cancers) have significantly increased the difficulty of any studies involving this cancer, because of broad variations in presentation and low study participant numbers [34]. The disease's rarity, combined with its lack of early symptoms and a range of possible alternative diagnoses, contributes to the challenge of identifying and investigating this deadly cancer [35]. Practically all cases are derived from glandular tissue, making them adenocarcinomas. Details of the gross morphology, histology, mode of spreading, and clinical manifestations are outlined in Figure 4 [36,37]. [36,37]

## Pathogenesis of Liver Fluke-Induced CCA

**Mechanical injury and fluke metabolic products.** Mechanical injury from the activities of feeding and migrating flukes contributes to biliary damage in the human host. Both oral and ventral suckers of the fluke hook onto the biliary epithelium, resulting in tissue damage even early in infection [38]. As the parasite matures, the lesion becomes more pronounced and ulcerates. Fluke eggs become entrapped in the periductal tissue through the ulcer and induce granulomatous inflammation around the eggs. The granulomata are readily visualized in experimental hamster infections and occasionally in human cases with bile duct obstruction.

The liver fluke secretes or excretes metabolic products, some of which are highly immunogenic, from the tegument and excretory openings into the bile or culture medium in vitro [39,40]. Apart from inducing host immune responses, the metabolic products themselves may be toxic to or interact with the biliary epithelium [41]. Indeed, mouse fibroblasts co-cultured with O. viverrini adult worms (but physically separated in Transwell plates) underwent more than 4-fold greater proliferation than cells in media alone [42].

Gene microarrays were recently used to explore transcriptional changes induced in these murine fibroblasts that were co-cultured with O. viverrini, and the fluke molecules induced over-expression of several mRNAs encoding growth-promoting proteins such as transforming growth factor [43]. Moreover, we observed that human CCA cell lines underwent excessive proliferation upon stimulation with O. viverrini soluble excretory/secretory (ES) products (B. Sripa et al., unpublished data). These studies clearly indicate that metabolic products of Opisthorchis contain mitogen-like activity and can induce cell proliferation. This is in agreement with earlier findings of hyperplasia of biliary epithelial cells in opisthorchiasis [38,44].

It is intriguing that O. viverrini would secrete a protein that promotes cell proliferation and eventually cancer, since the benefit to the parasite is unclear. One possibility might be that over-production of biliary cells may provide an abundant food source for the flukes, which graze on epithelial cells and/or their secreted mucins, though it has been proposed that C. sinensis ingests host blood through the damaged mucosa [12].

**Immunopathology.** It has long been suspected that host immune responses and immunopathologic processes mediate hepatobiliary damage in opisthorchiasis [38,45,46]. Sripa and Kaewkes [40] showed that inflammation around infected hamster bile ducts was a consequence of the host's cellular response to Opisthorchis antigens. Marked infiltration of inflammatory cells at the periportal areas of infected hamster liver was associated with the presence of parasite antigens in the bile duct epithelium as detected by immunohistochemistry. Small bile ducts, the first-order ducts (where flukes do not reside because the diameter of the ducts is too small), were also positive for O. viverrini antigens and were markedly inflamed. Opisthorchis antigens were also observed in macrophages, epithelioid cells, and giant cells of the granuloma [40].

Biliary cell damage induced by O. viverrini likely also stems from the actions of oxygen radicals such as nitric oxide (NO) released from effector cells activated by inflammatory cytokines [47,48]. These radicals can induce oxidative DNA damage to the infected biliary epithelium. Moreover, excess NO and other reactive oxygen intermediates produced by inflammatory cells during infection might exert direct cytotoxic and mutagenic effects and cause increased cell proliferation [49–51]. Increased formation of 8-nitroguanine (8-NO_2_-G) and 8-oxo-7,8-dihydro-2′-deoxyguanosine (8-oxodG) is associated with various pathological conditions [52,53], and 8-oxodG is considered to be mutagenic.

Recently, we reported diffuse nitrative and oxidative DNA damage (8-nitroguanine and 8-oxodG) in biliary epithelium of hamsters infected with O. viverrini [48], and these DNA lesions still remained in the epithelium at least 180 days post-infection. Moreover, repeated infections with liver fluke result in enhanced biliary DNA damage [54]. This may be explained by the fact that repeated infection increased inducible nitric oxide synthase expression in the bile duct epithelium. The DNA damage in infected biliary cells is proven to be a result of the inflammatory response caused by O. viverrini because 8-nitroguanine and 8-oxodG disappeared after praziquantel treatment [55]. Usually genotoxic events with DNA damage lead to either DNA-mismatch repair mechanisms or, if the damage is beyond repair, to cell death through apoptosis. NO not only induces DNA damage but has been reported to mediate DNA repair inhibition [56,57]. Moreover, NO has also been demonstrated to inhibit apoptosis downstream of cytochrome c [58]. All of these manifestations facilitate carcinogenesis.

Characterisation of the host immune responses—including the cytokine phenotypes—to O. viverrini and their roles in immune/inflammatory cell-induced tissue damage, as well as their relationship with host susceptibility, will likely yield a more complete understanding of the immunopathologic process involved in liver fluke–induced CCA.

**Figure 5:**
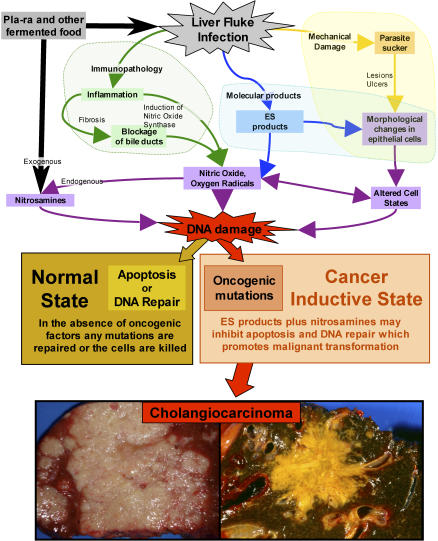
Proposed Mechanisms of Opisthorchis-Derived CCA Initiation The three proposed pathways linking the parasite to CCA initiation are mechanical damage (in yellow), molecular products (in blue), and immunopathology (in green). Combined, these mechanisms result in several common elements (purple) that all lead to DNA damage. The inhibition of a normal DNA damage response is the final oncogenic factor proposed to dramatically increase the likelihood of a malignant transformation. This invariably leads to progression of CCA. (Adapted from [10].) The photographs underneath the schematic show two livers with advanced CCA.

**Multi-factorial aetiology of CCA.** Liver fluke–induced CCA is therefore generally accepted to be the result of chronic inflammation [53,58,59]. Several mechanisms by which O. viverrini infection may enhance cholangiocarcinogenesis have been proposed above, and are summarised in Figure 5. Indeed, it is almost certain that a combination of those pathologies described above (mechanical damage, parasite secretions, and immunopathology) culminate in CCA after chronic infection with O. viverrini. The primary pathologic change, i.e., epithelial desquamation, may be due to mechanical irritation caused by the liver fluke and/or its metabolic products. However, immunopathologic processes may contribute to the long-standing hepatobiliary damage. During liver fluke infection, inflammation, periductal fibrosis, and proliferative responses, including epithelial hyperplasia, goblet cell metaplasia, and adenomatous hyperplasia, may represent predisposing lesions that enhance susceptibility of DNA to carcinogens [11,60]. Several *N*-nitroso compounds and their precursors occur at low levels in fermented food such as preserved mud fish paste, *pla-ra*, a condiment that is a ubiquitous component of the cuisine of northeastern Thailand and Laos.

Indeed, it has been hypothesised that *N*-nitroso compounds (e.g., nitrosamine) are a primary carcinogen leading to CCA in humans in this region [61,62]. We recently reported dissimilar gene expression profiles in CCA tissues from patients from sites endemic for O. viverrini in Thailand versus non-endemic areas in Japan [63]. Of particular interest was the selective up-regulation in Thai patients with CCA of genes involved in xenobiotic metabolism, implying that these genes may be involved in the detoxification of possible carcinogens such as nitrosamines [63]. CCA can be reliably induced in hamsters experimentally infected with O. viverrini and exposed to sub-carcinogenic doses of nitrosamine, but not in liver fluke–infected hamsters not exposed to nitrosamine [11].

Apart from exogenous carcinogens, however, endogenous nitrosation caused by liver fluke infection has also been investigated in animals and humans. Experimental Opisthorchis infection in hamsters can induce NO synthase expression by immune effector cells in inflamed areas surrounding the bile ducts and increased endogenous nitrosation of thiazolidine-4-carboxylic acid (thioproline) [64]. Several human studies suggest that infected people have a higher endogenous nitrosation potential than uninfected people [65,66]. Restricted diet studies clearly demonstrated an increase in endogenous generation of NO and *N*-nitroso compounds as indicated by increased levels of plasma, urinary nitrate and nitrite, and by nitrosation of proline and thioproline among infected men compared to members of an uninfected control group [25]. Moreover, the difference was specifically abolished by praziquantel treatment and by co-administration of ascorbic acid with proline. Enhanced immune responses to O. viverrini are also associated with an increase of endogenous nitrosation in infected individuals [67]. Both exogenous and in situ nitrosamine formation may lead to DNA alkylation and deamination in predisposed and inflamed tissues [49–51].

Apoptosis is rarely observed in either O. viverrini–infected hamster liver or in biliary cells that are co-cultured with flukes in vitro (B. Sripa et al., unpublished data). This may be explained by the mechanisms described above and/or the effect of bile acids [58]. Elevation of bile acids has also been reported in opisthorchiasis [68]. Bile acids are potent tumour promoters and have also been shown to block protein degradation of myeloid cell leukemia protein 1 (MCL-1), a potent anti-apoptotic protein of the BCl-2 family, via activation of *EGFR* signalling [69]. Anti-apoptosis has been described in infection-associated cancers such as those caused by Helicobacter pylori [70,71]. In the case of O. viverrini infection, DNA damage is caused in biliary epithelial cells while apoptotic mechanisms are dysregulated, resulting in genetic alterations which may become fixed, leading to malignant transformation.

## Implications for Improving Public Health

From a public health perspective, thorough cooking of the fish hosts efficiently blocks infection with these parasites. In this regard, liver fluke–associated CCA is preventable by changes in eating habits. Unfortunately, age-old culinary preferences for uncooked dishes such as *koi-pla* (Figure 1) do not readily allow for this possibility. Moreover, fish farming of grass carp and other susceptible species in ponds that are routinely contaminated by untreated sewage has resulted in the establishment of infection in fish populations at large, which, along with the involvement of animal reservoir hosts, makes control of liver fluke infection even more challenging [13,72]. Nonetheless, given this extraordinary linkage between a metazoan parasite and a tumour, characterisation of the nature and action of carcinogens of O. viverrini or C. sinensis may provide fundamental insights into carcinogenesis at large.

In the short term, enhanced knowledge of pathogenesis, particularly inflammation and its associated DNA damage—both nitrative and oxidative—may assist in disease control, provided the at-risk populations can be educated of the elevated risk of CCA from repeated infections. Also, dietary changes that include natural products rich in anti-oxidants may provide enhanced protection against CCA. In the longer term, it should be feasible to develop specific and sensitive techniques for detection of oxidative damage markers in serum, urine, or faeces for early cancer screening of the at-risk populations. We and others are now actively engaged in this endeavour.

## Supporting Information

Alternate Language Summary S1 A summary of the article translated into Thai by BS (29 KB DOC).

## Abbreviations

CCA: cholangiocarcinoma
ES: excretory/secretory
HCC: hepatocellular carcinoma
NO: nitric oxide
