# Supplementary material for: Liver Fluke Induces Cholangiocarcinoma
Source: PLoS Med. 2007 Jul 10;4(7):e201. doi: 10.1371/journal.pmed.0040201 (PMC1913093; doi:10.1371/journal.pmed.0040201)
Supplement: Supplementary file 1 [file pmed.0040201.sd001.doc]

**พยาธิใบไม้ตับกับการเหนี่ยวนำให้เกิดมะเร็งท่อน้ำดี**

โรคพยาธิใบไม้ตับที่เกิดจากการติดเชื้อพยาธิ *Opisthorchis viverrini*, *Opisthorchis felineus* และ *Clonorchis sinenesis* ยังเป็นปัญหาด้านสาธารณสุขที่สำคัญของภูมิภาคเอเชียตะวันออกเฉียงใต้ เอเชียตะวันออก ยุโรปตอนกลางและตะวันออก พยาธิใบไม้ตับเป็นโรคติดเชื้อเรื้อรังทำให้เกิดพยาธิสภาพของตับและท่อทางเดินน้ำดีหลายอย่างรวมทั้งมีความสัมพันธ์กับการเกิดมะเร็งท่อน้ำดีด้วย โดยเฉพาะพยาธิใบไม้ตับชนิด *Opisthorchis viverrini* ซึ่งพบระบาดมากในประเทศไทยและประเทศในเอเชียตะวันออกเฉียงใต้ถูกจัดให้อยู่ในกลุ่มพยาธิก่อมะเร็ง (Group 1, carcinogenic to human) เพราะมีหลักฐานทั้งทางระบาดวิทยาและการศึกษาในสัตว์ทดลองว่ามีความสัมพันธ์กับการเกิดมะเร็งท่อน้ำดีมากที่สุดในบรรดาพยาธิใบไม้ตับที่กล่าวมาข้างต้น พยาธิใบไม้ตับทำให้เกิดพยาธิสภาพในตับและท่อน้ำดีด้วยหลายกลไก เช่น จากการกัด-ดูดของ sucker พยาธิ และสารที่คัดหลั่งจากตัวพยาธิ มีหลักฐานชัดเจนว่าสารที่หลั่งจากตัวพยาธิทำให้นำไปสู่การเหนี่ยวนำให้มีเซลล์อักเสบมาตอบสนองในบริเวณที่มีพยาธิหรือโมเลกุลของพยาธิ นอกจากนี้ยังพบว่าสารที่คัดหลั่งจากตัวพยาธินี้ยังสามารถกระตุ้นทำให้เซลล์เยื่อบุท่อน้ำดีแบ่งตัวเพิ่มจำนวนได้มากขึ้น ในขณะที่มีการอักเสบจากการติดเชื้อนี้ก็จะมีการสร้างสารอนุมูลอิสสระจากเซลล์อักเสบออกมามากทั้งชนิดออกซิเจนและไนโตรเจน ซึ่งนำไปสู่การทำลายสารพันธุกรรมของเซลล์เยื่อบุท่อน้ำดีที่เรียกว่า oxidative and nitrative DNA damage	มีข้อมูลที่น่าตกใจคือยิ่งได้รับการติดเชื้อซ้ำบ่อยๆ ยิ่งทำให้มีการทำลายสารพันธุกรรมมากขึ้น การสารพันธุกรรมของร่างกายที่ถูกทำลายไปนี้ตามปกติร่างกายจะมีการซ่อมแซมให้กลับมาดีดังเดิมได้ หรือหากซ่อมแซมไม่ได้ร่างกายก็จะมีระบบกำจัดออกด้วยการตายแบบ apoptosis แต่ในกรณีของการทำลายสารพันธุกรรมจากการอักเสบนี้จะมีสารอนุมูลอิสสระชนิดหนึ่งคือ ไนตริคออกไซด์ ซึ่งจะไปยับยั้งการซ่อมแซมสารพันธุกรรม นอกจากนี้ยังมีรายงานว่ากรดน้ำดี ซึ่งพบสูงขึ้นในโรคติดเชื้อพยาธิใบไม้ตับ และ สารคัดหลั่งจากตัวพยาธิ มีฤทธิ์ต่อต้าน apoptosis ด้วย ดังนั้นเมื่อเซลล์เยื่อบุท่อน้ำดีมีสารพันธุกรรมถูกทำลายและไม่สามารถซ่อมแซมตัวเองหรือเกิด apoptosis ได้ แถมยังถูกกระตุ้นให้แบ่งตัวต่อได้ด้วยสารคัดหลั่งจากพยาธิ เซลล์นี้ก็จะมีการเปลี่ยนแปลงของสารพันธุกรรมผิดปกติแบบถาวร และนำไปสู่การเกิดมะเร็งท่อน้ำดีในที่สุด การได้รับทราบข้อมูลกลไกการเกิดมะเร็งท่อน้ำดีที่เกิดร่วมกับการติดเชื้อพยาธิใบไม้ตับนี้ ทำให้นักวิทยาศาสตร์สามารถศึกษาหาแนวทางที่จะป้องกันการเกิดโรคมะเร็งชนิดนี้ได้ เช่น ให้ความรู้เรื่องไม่กินปลาดิบซ้ำซาก	ให้กินอาหารที่มีสารต้านอนุมูลอิสสระ หรือ วิจัยหาหนทางที่จะตรวจสารบ่งชี้ชีวภาพ (Biomarker) ที่จำเพาะกับการทำลายสารพันธุกรรมเพื่อคัดกรองผู้ที่มีความเสี่ยงในการเป็นมะเร็งในพื้นที่ระบาดของพยาธินี้ ได้
